# High infectious disease burden as a basis for the observed high frequency of asymptomatic SARS-CoV-2 infections in sub-Saharan Africa

**DOI:** 10.12688/aasopenres.13196.1

**Published:** 2021-01-13

**Authors:** Kwadwo Asamoah Kusi, Augustina Frimpong, Frederica Dedo Partey, Helena Lamptey, Linda Eva Amoah, Michael Fokuo Ofori

**Affiliations:** 1 Department of Immunology, Noguchi Memorial Institute for Medical Research, College of Health Sciences, University of Ghana, Accra, Ghana

**Keywords:** SARS-CoV-2, COVID-19, immunity, tolerance, trained immunity, Africa

## Abstract

Following the coronavirus outbreaks described as severe acute respiratory syndrome (SARS) in 2003 and the Middle East respiratory syndrome (MERS) in 2012, the world has again been challenged by yet another corona virus, named severe acute respiratory syndrome coronavirus 2 (SARS-CoV-2). SARS-CoV-2 infections were first detected in a Chinese Province in December 2019 and then declared a pandemic by the World Health Organization in March 2020. An infection caused by SARS-CoV-2 may result in asymptomatic, uncomplicated or fatal coronavirus disease 2019 (COVID-19). Fatal disease has been linked with the uncontrolled “cytokine storm” manifesting with complications mostly in people with underlying cardiovascular and pulmonary disease conditions. The severity of COVID-19 disease and the associated mortality has been disproportionately lower in Africa and Asia in comparison to Europe and North America in terms of number of cases and deaths. While persons of colour who live in Europe and North America have been identified as a highly susceptible population due to a combination of several socioeconomic factors and poor access to quality healthcare, this has not been the case in sub-Saharan Africa where inhabitants are even more deprived concerning the said factors. On the contrary, sub-Saharan Africa has recorded the lowest levels of mortality and morbidity associated with the disease, and an overwhelming proportion of infections are asymptomatic. This review discusses the most probable reasons for the significantly fewer cases of severe COVID-19 disease and deaths in sub-Saharan Africa.

## Background

The 2019 novel human coronavirus, named severe acute respiratory syndrome coronavirus 2 (SARS-CoV-2), is one of seven coronaviruses that cause respiratory and intestinal diseases in humans ^1^. SARS-CoV-2 causes coronavirus disease 2019 (COVID-19) and this respiratory infection was declared a global pandemic in March 2020 ^2^. The novel virus infects the host by using its surface (S) protein to interact with the host angiotensin-converting enzyme 2 (ACE2) receptors found in the lungs and other organs and subsequently fuses with the host cell membrane ^3– 5^. Clinical symptoms of SARS-CoV-2 infection generally include fever, headache, loss of one’s sense of smell, malaise, sore throat and muscular pain, which appear within 2 – 14 days post-infection. These symptoms are usually followed by a dry cough and difficulty in breathing, and can rapidly progress to more life-threatening events such as respiratory failure and acute respiratory distress syndrome ^2, 6^. Infected persons may not necessarily exhibit all of these symptoms, but do exhibit a combination of these symptoms.

COVID-19 has exposed weaknesses in health systems globally and pointed to the need to strengthen these health systems and also put a significant emphasis on disease prevention. Emerging literature shows that there are wide geographic and demographic differences in the symptoms and presentation of the disease. The most at-risk groups include older persons above 60 years, the immunocompromised and persons of all ages who have some underlying conditions including diabetes, high blood pressure and other cardiovascular conditions ^7^. There are, however, a significant number of infected persons who remain asymptomatic or develop only mild self-limiting symptoms ^8^. For example, while an estimated 80% of SARS-CoV-2 infections are asymptomatic or result in mild disease, the remaining 20% of patients can become severely ill, although the majority in this latter category may have co-morbidities with conditions such as diabetes and hypertension ^6, 7^. Mortality is therefore disproportionately high in infected persons with underlying comorbidities.

## Association between race and SARS-CoV-2 infection outcomes

Current evidence from Europe and the Americas suggests that people of African descent living in these areas are more susceptible to the severe forms of COVID-19 and more often die from COVID-19 related causes compared to other races, especially Caucasians ^9, 10^. The high levels of morbidity and mortality in persons of African descent living in Europe and the Americas have been partly attributed to the relatively higher incidence of co-morbid conditions and low socioeconomic status resulting in low access to appropriate healthcare and good housing, high housing density and limited access to healthy foods ^10, 11^. This greater susceptibility of people of African descent is, however, in sharp contrast with the growing observation that a significant majority of SARS-CoV-2 infections in sub-Saharan Africa are asymptomatic or only develop very mild symptoms. An intriguing factor to consider here is that the predisposing socioeconomic factors that have been associated with the greater susceptibility of people of African descent who are resident in Europe and the Americas are even more pronounced in sub-Saharan Africa. Therefore, neither these socioeconomic factors nor genetic factors can explain the observed significant disparities in SARS-CoV-2 infection outcomes between Africans living in sub-Saharan Africa and those elsewhere.

At the population level, SARS-CoV-2 infections in Europe and the Americas have resulted in a significantly higher number of deaths compared to cases in sub-Saharan Africa ^12^. While Africa’s younger population and hence relatively lower prevalence of underlying conditions ^13^ that have been identified as COVID-19 risk factors may be an important explanatory variable, this alone cannot fully explain the observed wide differences in COVID-19 case severity and mortality between sub-Saharan Africa and the developed world. There is therefore an urgent need to unravel the aetiologic basis of SARS-CoV-2 infection and progression to disease states in different populations. Also, within a given population, it is essential to identify factors aside from co-morbidities that account for why some individuals become severely ill while others only show mild symptoms or remain asymptomatic throughout the infection.

## Immunity and immunopathology in COVID-19 patients

Infection with SARS-CoV-2 elicits both innate and adaptive immune responses, although the underlying mechanisms are just beginning to be dissected. Non-specific defense molecules secreted by several immune cells upon stimulation by pathogen antigens result in the induction of inflammation, which is a natural immune response that is required to control the spread and multiplication of the pathogen. Highly activated cells of the innate immune system, including macrophages, neutrophils and dendritic cells have been shown to predominate in the lung tissues of COVID-19 patients ^6, 14^. Dendritic cells and macrophages express toll-like receptors that are used in sensing viral RNA and lead to the activation of the nuclear factor kappa B (NF-κB) pathway and the induction of pro-inflammatory cytokines. Cytokines such as interleukin-1 beta (IL-1β) are important in the development of the virus-induced inflammation associated with disease severity ^15, 16^. Excessive inflammation, however, can result in collateral damage to normal host cells. In severely sick COVID-19 patients, there seems to be an infection-related disproportionate increase in the numbers of innate cells such as neutrophils, monocytes and macrophages, relative to the number of lymphocytes ^6, 17– 19^. It has also been observed that there is a heightened expression of inflammatory molecules in the lung tissues of COVID-19 patients compared to regular pneumonia patients and healthy controls ^14, 20^. Our current understanding of life-threatening disease aetiology relates to the development of severe disease symptoms as a result of the induction of a cytokine storm which causes/aggravates the observed lung pathology ^21, 22^. The non-specific immune responses, mostly from innate immune cells, are therefore more likely to be associated with the observed immunopathology.

## Pathogen-induced immunological tolerance to inflammation

Clinical pathology associated with some infectious diseases can be traced to a dysregulation of the immune responses that are elicited against the infecting pathogens. Persistent or chronic exposure of persons to these infectious pathogens, however, causes a state of immunological tolerance to pathogen-induced inflammation ^23^. For disease conditions such as malaria, the inflammatory immune response mounted against the parasite can result in immunopathology if not properly regulated ^24, 25^. There is, however, growing evidence that in areas with sustained high transmission, persons with increased or frequent exposure to malaria parasites develop a high tolerance threshold to inflammation compared to persons with a low parasite burden ^26^. Adults who have experienced repeated infections are also more tolerant to high parasitaemia compared to young children ^27^. There is also evidence for the induction of immunological tolerance by other pathogens, including helminths, bacterial and viral infections ^28– 32^ . For lung infections, the induction and relevance of immunological tolerance to the survival of infected patients have been reviewed recently ^33^. During SARS-CoV-2 infection, severe clinical symptoms including pulmonary pneumonia and bronchitis which can ultimately lead to acute respiratory distress syndrome and respiratory failure ^2, 6^ are aetiologically associated with an unregulated production of pro-inflammatory cytokines in lung tissues which results in a cytokine storm ^22^. In persons whose systems have been primed by repeated exposure to infectious agents and are hence able to effectively regulate the production of high levels of pro-inflammatory mediators, SARS-CoV-2 infections may not exhibit the same cytokine storm features as is seen in persons with limited exposure to infectious agents. The capacity to exhibit greater immunological tolerance to subsequent infections therefore protects against the development of severe clinical symptoms as a result of SARS-CoV-2 infections

## BCG and the concept of trained immunity

BCG is a live attenuated vaccine that is used for the prevention of tuberculosis (TB), and this vaccine has the attenuated bacterium *Mycobacterium bovis* as the vaccine agent. Bacterial cell wall components in BCG collectively called pathogen-associated molecular patterns (PAMPs) are known to trigger Toll-like receptors on cell types such as macrophages, neutrophils and dendritic cells at the sites of injection to induce potent, non-specific pro-inflammatory responses ^34– 37^. Following vaccination, the live *Mycobacterium* is internalized by dendritic cells and can live up to two weeks within these cells during which specific BCG antigens have been shown to trigger the prolonged production of the pro-inflammatory mediators including tumor necrosis factor, IL-6 and IL-1-β which plays a vital role in anti-viral immunity ^37– 40^. Bickett *et al.* also show in a mouse model that BCG is a potent innate immune regulator that elicits long-lived T cell-independent protection against pulmonary TB ^41^. Thus, BCG vaccination generally increases the homeostatic threshold of local inflammation in the lungs, and this may make SARS-CoV-2-infected persons more tolerant to the virus-induced local inflammation in the lungs.

There is growing evidence that innate immune cells can be primed by PAMPS from one pathogen to develop into a memory phenotype that can recall responses to similar PAMPs from other pathogens ^34, 35, 41^. This phenomenon, called trained immunity, enables these innate cells to mount a “secondary” response to PAMPs from other pathogens and thereby protect against infections caused by these other pathogens. There is also the suggestion that the innate immune cells rely on epigenetic reprograming to obtain memory from previous exposure to an infectious agent. Thus, aside the innate immune cells being trained, they also retain memory in hematopoietic stem cell precursors in the bone marrow, which results in establishing long-lasting memory after several exposures to other infections ^42^.

It has already been shown that BCG vaccination in children has a significant effect in reducing about 50% of the mortality associated with the incidence of sepsis and other respiratory infections ^43, 44^. This mechanism of protection has been strongly linked to the ability of the innate cells to elicit a polarized pro-inflammatory immune response during non-specific immune reactivation ^40, 45^. This non-specific immune response against BCG vaccination has been shown to be protective against other infections and tumors and associated with trained immunity ^46^. It has also been observed that SARS-CoV-2 infected persons who have been vaccinated with BCG have some level of protection against severe disease development ^46, 47^. This position is further affirmed by the observation that African countries such as South Africa, which have a relatively lower infectious disease burden compared to most other sub-Saharan countries have reported generally higher numbers of severe SARS-CoV-2 cases and deaths ^48^. Therefore, as seen with BCG vaccination, the mechanisms underlying protection against severe COVID-19 disease could be related to the development of trained immunity against natural Mycobacterial infections in highly exposed populations.

Globally, TB is most prevalent in sub-Saharan Africa and Southern Asia, and similar effects can be expected to result from natural *Mycobacterium* infections. Indeed, Southern Asia has also recorded very few SARS-CoV-2 related severe disease and deaths ^49^. Countries such as India, China, Indonesia, Pakistan and the Philippines that have the world’s highest TB prevalence have reported significantly fewer cases of severe COVID-19 requiring hospitalization and fewer COVID-19 related deaths compared to Central Europe and North America ^50, 51^. This observation thus further affirms the role of increased pathogen exposure in protection against the novel coronavirus. Aside from BCG, cross-protection against COVID-19 and other infectious diseases has recently been postulated for the live oral polio vaccine, and the mechanisms of protection are most likely to be related ^52^.

In addition to the above, a recent study by Tso and colleagues examining pre-COVID-19 plasma has demonstrated that individuals from Tanzania and Uganda harbor significantly high human coronavirus (HCoV)-specific antibodies that cross-react with SARS-COV-2 nucleocapsid and spike proteins compared to US volunteers ^53^. The high disease burden in Sub-Saharan Africa could lead to prior exposure to other widely circulating human coronaviruses where immunity acquired against other HCoVs protects against the novel COVID-19. It is worth noting that although the HCoV antibodies were shown to cross-react with SARS-COV-2, the functional abilities of the cross-reactive antibodies and whether they are protective remain unknown ^54^. A larger pre-COVID-19 sample size with longitudinal sampling points will be needed for comprehensive analysis of cross-reactive B cells and T cell function ^54^ and their correlation with COVID-19 clinical outcomes and disease epidemiology.

## Concluding remarks

The SARS-CoV-2 pandemic has so far resulted in significant numbers of deaths in the developed world and the same was expected to happen in sub-Saharan Africa due to the generally weak health systems amongst other factors. This has, however, not been the case, and the available literature suggests that the highly infectious disease burden on the African continent could be a very significant factor that can explain the high proportion of asymptomatic SARS-CoV-2 infections in sub-Saharan Africa. The high infectious disease burden and frequent exposure to infectious agents may mediate the asymptomatic SARS-CoV-2 infection status in two major ways. The first is through the induction of immunological tolerance and the consequent resistance to the development of immunopathology. Thus, although there is the induction of inflammatory responses against SARS-CoV-2 in infected persons, these responses may be well balanced homeostatically such that they do not induce the pathology that is known to be associated with severe infections, and which predispose to death.

 Second is the induction of trained immunity by previous infections with other lung pathogens such as TB, which is very prevalent in Africa and South-East Asia. In addition to the evidence presented for the immunological tolerance induction mechanism, the contribution of a trained immunity mechanism to the current observations cannot be easily overlooked. Either way, the higher burden of infectious diseases remains the common denominator as the most probable reason for the observed lower numbers of severe cases of COVID-19 disease and related deaths in Africa. The low prevalence of severe cases and mortality notwithstanding, African countries need to be more vigilant and enforce the COVID-19 prevention protocols to avoid being overwhelmed should more virulent forms of the virus emerge. The recent emergence of mutant viral forms with increased transmissibility in the United Kingdom and South Africa ^55^ is a clear testament to this, albeit these new variants have not been necessarily shown to be more virulent.

## Data availability

### Underlying data

No data are associated with this article
