# Supplementary material for: High infectious disease burden as a basis for the observed high frequency of asymptomatic SARS-CoV-2 infections in sub-Saharan Africa
Source: AAS Open Res. 2021 Oct 18;4:2. Originally published 2021 Jan 13. [Version 3] doi: 10.12688/aasopenres.13196.3 (PMC8524298; doi:10.12688/aasopenres.13196.3)
Supplement: Peer review history

# Peer review: Reviewer response for version 3

**DOI:** 10.21956/aasopenres.14445.r28954

**Published:** 2021-10-18

**Authors:** Jean-Michel Heraud

**Affiliations:** 1 Virology Unit, Institut Pasteur de Dakar, Dakar, Senegal

I do not have any more comments regarding this latest version.

Is the review written in accessible language?

Yes

Are all factual statements correct and adequately supported by citations?

Yes

Are the conclusions drawn appropriate in the context of the current research literature?

Partly

Is the topic of the review discussed comprehensively in the context of the current literature?

Yes

Reviewer Expertise:

Virology, Public health

I confirm that I have read this submission and believe that I have an appropriate level of expertise to confirm that it is of an acceptable scientific standard.

# Peer review: Reviewer response for version 2

**DOI:** 10.21956/aasopenres.14364.r28914

**Published:** 2021-10-07

**Authors:** Jean-Michel Heraud

**Affiliations:** 1 Virology Unit, Institut Pasteur de Dakar, Dakar, Senegal

In this interesting manuscript, the authors postulate some hypotheses explaining the relative lower impact in terms of the disease burden associated with SARS-CoV-2 in sub-Saharan Africa. The manuscript is well written and easy to read. The pillar of the observed low number of severe COVID-19 cases and associated mortality is explained, according to authors, to particular immunity of African populations which, the immune systems have been “trained” by the microbial environment inherent of African setting.

 This hypothesis is possible and acceptable but I doubt that it is this typical characteristic that is the basis for the observed high frequency of asymptomatic and I would appreciate some addition from authors that proposed other potential co-factors.

 For that reason, I have some minor revisions before the indexing of the manuscript:

1. I don’t think that the title is fully relevant. Indeed, the authors assumed that frequency of asymptomatic is higher in sub-Saharan Africa. Although it can be true, to my knowledge there is no clear demonstration and it is well known for other diseases that patients in particular amongst adults are less prompt to consult for mild and paucisymptomatic clinical presentation, due to several factors (behaviour, limited access to health care, low income, etc.). I would rather prefer a title that mentioned lower severe SARS-CoV-2 infections. I also prefer to mention the “particular microbial environment” in Africa instead of the High infectious disease burden since we can’t be sure that some diseases could explain all and “protect” individuals from severe COVID-19. Indeed, HIV is highly prevalent in Africa and has no protective effect.
2. The paragraph regarding cross protection from previous HCoV infection should be included in the section “Pathogen-induced immunological tolerance to inflammation".
3. In the discussion, I would like the authors to clearly mention that their hypothesis is one of the explanation and the relative lower burden of COVID-19 in Africa is probably multi-factorial (lower population density and mostly rural, climatic drivers? few elderly living in residential homes …). Moreover, as mentioned by the authors, it is important to highlight the need to conduct studies aiming at estimating the real “toll” and burden of COVID-19 in African settings. Authors could mention recently published serological studies from sub-Saharan African (Kenya, Madagascar, …)

Is the review written in accessible language?

Yes

Are all factual statements correct and adequately supported by citations?

Yes

Are the conclusions drawn appropriate in the context of the current research literature?

Partly

Is the topic of the review discussed comprehensively in the context of the current literature?

Yes

Reviewer Expertise:

Virology, Public health

I confirm that I have read this submission and believe that I have an appropriate level of expertise to confirm that it is of an acceptable scientific standard, however I have significant reservations, as outlined above.

# Peer review: Reviewer response for version 2

**DOI:** 10.21956/aasopenres.14364.r28647

**Published:** 2021-08-10

**Authors:** Marco Bongiovanni

**Affiliations:** 1 Internal Medicine Unit, Department of Medicine, Ospedale di Circolo di Rho, ASST Rhodense, Milan, Italy

The authors implemented into their paper my suggestions, therefore the article can now be accepted.

Is the review written in accessible language?

Yes

Are all factual statements correct and adequately supported by citations?

Yes

Are the conclusions drawn appropriate in the context of the current research literature?

Partly

Is the topic of the review discussed comprehensively in the context of the current literature?

Yes

Reviewer Expertise:

Infectious Diseases, COVID-19

I confirm that I have read this submission and believe that I have an appropriate level of expertise to confirm that it is of an acceptable scientific standard.

# Peer review: Reviewer response for version 1

**DOI:** 10.21956/aasopenres.14308.r28539

**Published:** 2021-05-12

**Authors:** Marco Bongiovanni

**Affiliations:** 1 Internal Medicine Unit, Department of Medicine, Ospedale di Circolo di Rho, ASST Rhodense, Milan, Italy

This is an interesting paper that evaluates and discusses the high prevalence of asymptomatic COVID-19 infections in Sub Saharan Africa. In my opinion, it should also discuss other points that can be explained by this observation.

1. Usually, people living in Sub Saharan Africa are young and it is well known that age is one of the most important predictors of mortality in COVID patients.
2. Another possible point to be discussed deeply is the possible absence of diagnosis especially in very poor countries with very limited health resources.
3. The impact of COVID-19 infection in large crowded cities and sparsely inhabited villages should be further differentiated.

I would ask the authors to discuss also these points in their conclusions.

Is the review written in accessible language?

Yes

Are all factual statements correct and adequately supported by citations?

Yes

Are the conclusions drawn appropriate in the context of the current research literature?

Partly

Is the topic of the review discussed comprehensively in the context of the current literature?

Yes

Reviewer Expertise:

Infectious Diseases, COVID-19

I confirm that I have read this submission and believe that I have an appropriate level of expertise to confirm that it is of an acceptable scientific standard, however I have significant reservations, as outlined above.

# Response

**Published:** 2021-05-28

**Authors:** Kwadwo Asamoah Kusi

**Affiliations:** Noguchi Memorial Institute for Medical Research, Ghana

We are grateful for the reviewer's comments as this has served as basis for us to improve upon the manuscript. We agree that the factors mentioned will all contribute to explaining the observation of high asymptomatic COVID-19 infections in Africa to some degree, our paper to identifies the high infectious disease burden as the major contributor to explaining these observations.

 We have however addressed the comments raised by the reviewer and made updates to other sections of the manuscript to reflect the current state of knowledge of the pandemic.
